# Effects of Neuromuscular Electrical Stimulation Training on Endurance Performance

**DOI:** 10.3389/fphys.2016.00544

**Published:** 2016-11-16

**Authors:** Menno P. Veldman, Julien Gondin, Nicolas Place, Nicola A. Maffiuletti

**Affiliations:** ^1^Center for Human Movement Sciences, University Medical CenterGroningen, Netherlands; ^2^Human Performance Lab, Schulthess ClinicZurich, Switzerland; ^3^Institut NeuroMyoGène, Université Claude Bernard Lyon 1, INSERM U1217, CNRS UMR 5310Villeurbanne, France; ^4^Institute of Sport Sciences, University of LausanneLausanne, Switzerland; ^5^Department of Physiology, Faculty of Biology and Medicine, University of LausanneLausanne, Switzerland

**Keywords:** neuromuscular electrical stimulation, skeletal muscle, muscle endurance, functional endurance, rehabilitation

## Introduction

Various electrical stimulation modalities are used as adjuvants to conventional training and rehabilitation programs to increase bodily function or to reduce symptoms, such as pain. One of these modalities, neuromuscular electrical stimulation (NMES), commonly refers to the transcutaneous application of electrical currents to a target muscle group with the objective to depolarize motor neurons and consequently elicit skeletal muscle contractions of substantial intensity (usually ranging from 10 to 60% of the maximal voluntary contraction). Because NMES can generate considerable muscle tension, it is frequently used as a strength training technique for healthy adults and athletes, but also as a rehabilitation tool to increase or preserve muscle function and mass in individuals with muscle weakness or patients who cannot perform voluntary contractions [e.g., patients suffering from chronic heart failure (CHF), chronic obstructive pulmonary disease (COPD), or critical illness; for reviews, see Roig and Reid, 2009; Sillen et al., 2009; Sbruzzi et al., 2010; Maddocks et al., 2011; Maffiuletti et al., 2011; Smart et al., 2013; Burke et al., 2016]. Under certain conditions, NMES training may also improve muscle oxidative capacity and result in a fast-to-slow muscle fiber type transition (Pérez et al., 2002; Gondin et al., 2011a), which could potentially enhance endurance performance. However, the relevance of such adaptations in skeletal muscle tissue for the translation to functional performance that is particularly important for sport and daily activities is not always self-evident, mainly because of the heterogeneity in study populations, NMES parameters, and outcome measures. Unfortunately, the bodies of literature that either focus on mechanistic (i.e., muscle endurance) or clinical (i.e., functional endurance) outcomes are often too disconnected. In this opinion paper, we aim to bring these bodies of literature together and discuss the impact of high- vs. low-frequency NMES training on muscle vs. functional endurance in healthy vs. clinical populations. As such, we focus on human studies that chronically applied NMES for at least 3 weeks in healthy persons and patients, and distinguish between the effectiveness of non-tetanic low-frequency NMES (that is usually administered continuously at frequencies close to 10 Hz) and tetanic high-frequency NMES (that is usually administered intermittently at frequencies close to 50 Hz) on muscle endurance and functional endurance. For clarity purposes, we refer to muscle endurance as the exercise-induced decline in voluntary or electrically-evoked force (Duchateau and Hainaut, 1988; Gondin et al., 2006) or the endurance time of a sustained single-joint contraction (Gondin et al., 2006). In contrast, we refer to functional endurance as the maximal oxygen consumption or workload (Pérez et al., 2002; Porcelli et al., 2012), the distance covered in a given time (e.g., 6-minute walk test) or the endurance time (Kim et al., 1995) for whole-body exercises such as walking and cycling. In the last section, we will provide some recommendations for better clinical use of NMES, and suggest potential directions for future research.

## Effects of NMES training on endurance performance in healthy subjects

**Figure 1:**
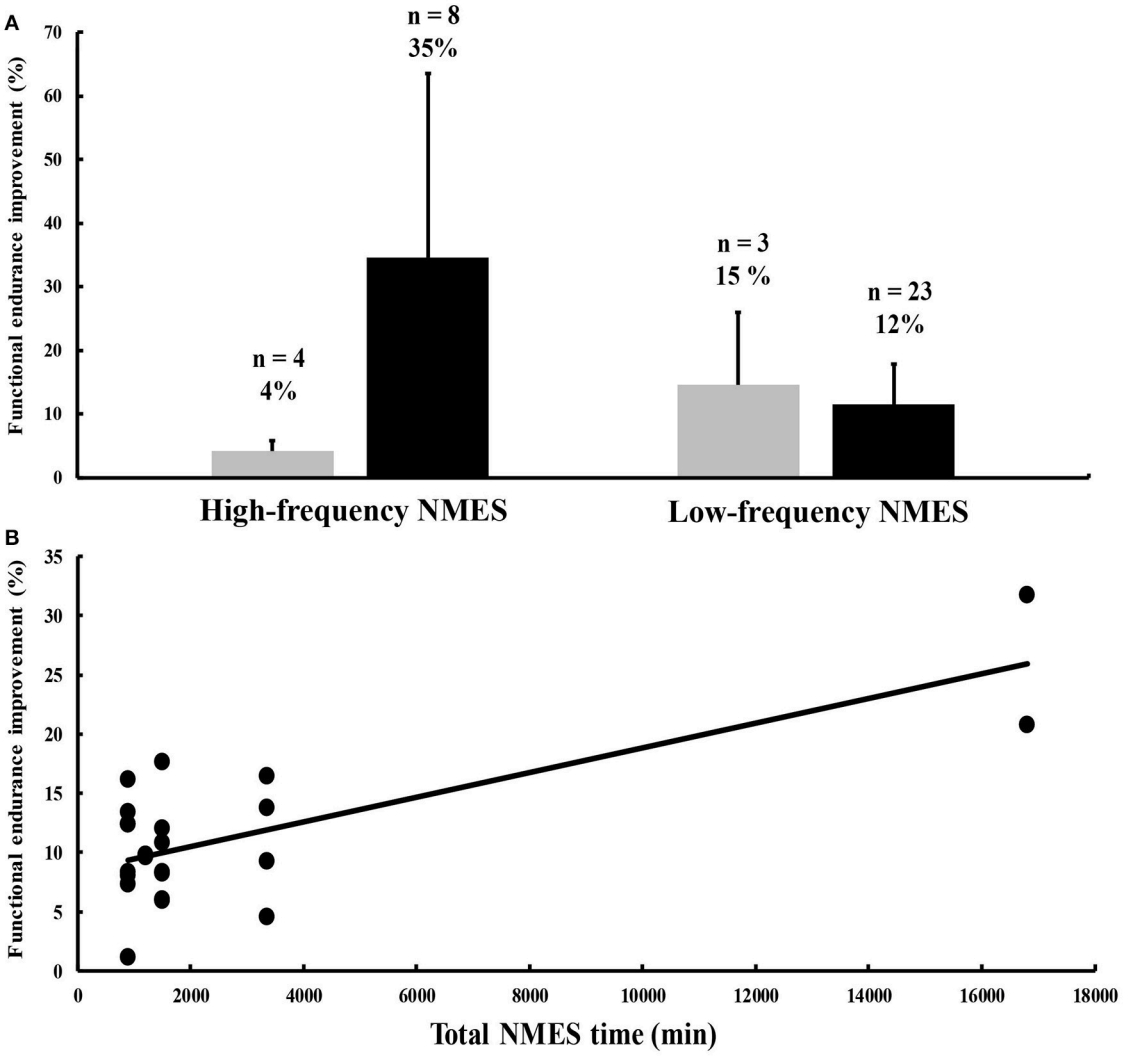
**(A)** Percent improvements in functional endurance after high- and low-frequency neuromuscular electrical stimulation (NMES) interventions in healthy subjects (gray histograms) and patient populations (black histograms). The numbers above each histogram represent the number of outcome measures the mean change was computed from, and the mean percent change of these outcome measures. Most of the data, which are not stratified by condition and type of outcome measure, were extracted from Sillen et al. (2009). Vertical bars denote one standard deviation. **(B)** Relationship between the total duration of NMES treatment on the x-axis and NMES training-induced percent improvements in functional endurance in patients suffering from chronic heart failure (CHF) on the y-axis (anecdotally, the *R*^2^ is 0.54). Data were obtained from eight different studies published between 2003 and 2006 (Harris et al., 2003; Nuhr et al., 2003; Eicher et al., 2004; Banerjee et al., 2005; Deley et al., 2005; Dobsák et al., 2006; Karavidas et al., 2006; LeMaitre et al., 2006).

High-frequency NMES training enhances muscle strength (for a review, see Gondin et al., 2011b), but may not affect (Duchateau and Hainaut, 1988) or even decrease muscle endurance, as demonstrated for example by a reduced ability to sustain a submaximal contraction (Gondin et al., 2006). High-frequency NMES training also appears to have a negligible influence on functional endurance, as illustrated for example by maximal oxygen consumption results (Figure 1A) (Pérez et al., 2002; Porcelli et al., 2012). These limited effects of high-frequency NMES training on endurance performance are somewhat surprising for different reasons. First, a single session of high-frequency NMES induces an exaggerated metabolic and cardiovascular stress when compared to torque-matched voluntary contractions (McNeil et al., 2006; Theurel et al., 2007) and high levels of muscle fatigue, mainly due to the motor unit recruitment pattern of NMES that is considerably different from voluntary contractions (for a review, see Bickel et al., 2011). Second, high-frequency NMES results in a fast-to-slow shift in fiber type distribution together with increased oxidative capacity and capillarization of the stimulated muscles (Pérez et al., 2002; Gondin et al., 2011a), i.e., adaptations that are characteristic of endurance training. However, methodological limitations pertaining to the definition and assessment of muscle or functional endurance complicate the interpretation of the data. Also, the evaluation of endurance for commonly-used muscles such as the quadriceps in normally functioning people may not be optimal as it likely suffers from ceiling effects. This suggestion is confirmed by the observation that high-frequency NMES training of abdominal muscles in healthy individuals resulted in substantial increases in abdominal strength and endurance time (Alon et al., 1987).

Data on the effects of low-frequency NMES training on muscle and functional endurance in healthy participants are relatively scarce. Three studies show improvements in functional endurance following low-frequency NMES training (e.g., improved work capacity and oxygen consumption at the anaerobic threshold), possibly mediated by adaptations in aerobic-oxidative metabolism and increased capillarization (Thériault et al., 1996; Nuhr et al., 2003; Miyamoto et al., 2016). Notwithstanding the limited data, the effects of low-frequency NMES training on functional endurance appear in general superior to those induced by high-frequency NMES training, despite the apparently similar fast-to-slow transition in fiber type distribution induced by the two NMES modalities (Thériault et al., 1996; Nuhr et al., 2003). Such divergent effectiveness of high- vs. low-frequency NMES training on endurance performance is likely caused by methodological differences between the two modalities. High-frequency NMES is usually applied intermittently and with higher current intensities in comparison with low-frequency NMES. Because of better current tolerance, low-frequency NMES sessions are generally considerably longer and can reach up to 240 min of continuous stimulation per day (e.g., Nuhr et al., 2003), vs. 20–30 min of intermittent high-frequency NMES per day (Gondin et al., 2011a). Such differences may have contributed to the greater increases in functional endurance observed after low- vs. high-frequency NMES training and strongly suggest that the long duration of low-frequency NMES sessions may be the most important parameter for increasing functional endurance.

In summary, little is known about the impact of NMES training on muscle and functional endurance in a healthy population. High-frequency NMES training has no influence or may even have a negative impact on muscle endurance while for functional endurance, low-frequency NMES training appears favorable, possibly because of the very long treatment sessions. Studies in healthy individuals that often targeted the quadriceps muscle may have suffered from ceiling effects. We speculate that NMES interventions on less-used muscle groups and with stronger study designs (large sample size, homogeneous subject characteristics, sham condition) will provide more insights into the real effectiveness of NMES training.

## Effects of NMES training on endurance performance in patient populations

The suggestion that less-used muscle groups are more prone to improvement is confirmed by findings obtained in patients with pathologies affecting muscle, pulmonary, and cardiovascular function. In general, NMES training protocols are more effective in patients compared to healthy individuals, at least for functional endurance (Figure 1A). In various patient groups such as COPD and CHF, NMES training improved muscle strength and respiratory function, and consequently, functional endurance as reflected by increased oxygen uptake and workload, longer endurance times and farther movement distances, often indexed by the 6-minute walk test (for reviews, see Roig and Reid, 2009; Sillen et al., 2009; Sbruzzi et al., 2010; Maddocks et al., 2011; Smart et al., 2013; Burke et al., 2016). In contrast, there are few data on adaptations in muscle endurance after high- or low-frequency NMES training in patient populations. To the best of our knowledge, only three studies reported improvements in muscle endurance in different patient groups following NMES interventions (Quittan et al., 2001; Doucet and Griffin, 2013; Erickson et al., 2016). Clinical trials that applied high- and low-frequency NMES training focused mostly on functional endurance because of its clinical relevance (Vaquero et al., 1998; Bourjeily-Habr et al., 2002; Neder et al., 2002; Harris et al., 2003; Nuhr et al., 2003; Eicher et al., 2004; Banerjee et al., 2005; Deley et al., 2005; Dobsák et al., 2006; Karavidas et al., 2006; LeMaitre et al., 2006; Vivodtzev et al., 2006, 2012; Dal Corso et al., 2007).

Figure 1A, which contains data from the 14 aforementioned clinical trials, shows more favorable effects of high-frequency over low-frequency NMES training on functional endurance. This non-comprehensive analysis is confounded by the disparate definitions of functional endurance, the absence of stratification for condition, and the heterogeneity in NMES parameters. For example, for some unknown reasons, five studies in patients suffering from COPD used exclusively high-frequency NMES and showed greater increases in functional endurance (43%) compared to nine studies conducted in CHF patients that were administered almost exclusively low-frequency NMES (12%). Nevertheless, the rate of improvement may also depend on the severity of the disease. For example, patients with severe CHF improved more (26%; Nuhr et al., 2003) compared to stable patients (10%; e.g., Deley et al., 2005) following a low-frequency NMES training program. More importantly, the total duration of the NMES treatment (i.e., the training volume) from eight different studies conducted on CHF patients seems to be positively related to the magnitude of the improvement in endurance performance (Figure 1B).

In summary, both high- and low-frequency NMES training can increase functional endurance in patient populations, while their influence on muscle endurance is largely unknown (probably because of the poor clinical relevance of this latter variable). The impact of NMES training protocols on endurance performance appears highly dependent on the type of disease, its severity, and the total exposure to the treatment.

## Conclusions

High- and low-frequency NMES training can increase functional endurance, with the magnitude of the effects being dependent on the initial condition. That is, inactive patients with advanced disease are more likely to benefit from NMES training than more active patients with stable symptoms and healthy individuals. The most likely explanation for this observation is that the quadriceps muscle, mostly targeted by NMES, is highly involved in several activities of daily living and is therefore less sensitive to improvements in active individuals. We therefore argue that NMES training is particularly useful for patients that are unable or unwilling to participate in daily activities or in regular physical exercise. Despite the heterogeneity of the studies in terms of study populations, NMES parameters, and outcome measures, we provide recommendations for clinical use and present ideas to increase treatment effectiveness. Although recent comparison studies did not show differences in the acute responses between high- and low-frequency NMES in both CHF (Sbruzzi et al., 2010) and COPD patients (Sillen et al., 2011), low-frequency NMES training seems to be particularly effective for patients with CHF, with longer treatment durations causing larger increases in functional endurance (Figure 1B), while for COPD patients, it is difficult to provide specific recommendations concerning high- vs. low-frequency NMES due to limited data.

Although the literature has provided us with many mechanistic and clinical insights into NMES training-induced effects on muscle and functional endurance, the trials conducted so far have some methodological limitations and need to be improved. First, there is a clear distinction between the trials in healthy participants and those in patient populations. While studies in healthy individuals focus on mechanistic parameters such as fiber type composition and capillarization and have few outcome measures that are clinically relevant, studies in patient populations merely focus on functional/clinical outcomes. While such distinctions are understandable from a patient-burden point of view, the mechanisms underlying NMES-induced adaptations may differ between diseased and healthy individuals. Therefore, randomized controlled trials are needed that compare high- and low-frequency NMES training programs for both lower and upper extremity muscle groups in patient populations vs. age- and gender-matched healthy controls. In addition, because the clinical applicability also depends on whether NMES-induced effects can still be observed after several months, follow-up measures should be included in future trials.

In conclusion, we propose here that both high- and low-frequency NMES training (and probably a combination of the two, depending on clinicians' needs) are potentially relevant to improve endurance performance, and that although their physiological effects are relatively well understood in healthy subjects, more evidence-based research is required to optimize NMES treatment protocols for various patient populations.

## Author contributions

All authors listed, have made substantial, direct and intellectual contribution to the work, and approved it for publication.

### Conflict of interest statement

The authors declare that the research was conducted in the absence of any commercial or financial relationships that could be construed as a potential conflict of interest.
